# Oxysterol derivatives Oxy186 and Oxy210 inhibit WNT signaling in non-small cell lung cancer

**DOI:** 10.1186/s13578-022-00857-9

**Published:** 2022-07-30

**Authors:** Liu-Ya Tang, Marie Spezia, Ting Chen, Jee-Hye Shin, Feng Wang, Frank Stappenbeck, Andres M. Lebensohn, Farhad Parhami, Ying E. Zhang

**Affiliations:** 1 Laboratory of Cellular and Molecular Biology, Center for Cancer Research, National Cancer Institute, National Institutes of Health, NIH, Building 37, RM 2056B, Bethesda, MD 20892 USA; 2 Max Biopharma. Inc, 2870 Colorado Avenue, Santa Monica, CA 90404 USA

**Keywords:** Oxysterol, WNT, Hedgehog, NSCLC

## Abstract

**Background:**

Developmental signaling pathways such as those of Hedgehog (HH) and WNT play critical roles in cancer stem cell self-renewal, migration, and differentiation. They are often constitutively activated in many human malignancies, including non-small cell lung cancer (NSCLC). Previously, we reported that two oxysterol derivatives, Oxy186 and Oxy210, are potent inhibitors of HH/GLI signaling and NSCLC cancer cell growth. In addition, we also showed that Oxy210 is a potent inhibitor of TGF-β/SMAD signaling. In this follow-up study, we further explore the mechanism of action by which these oxysterols control NSCLC cell proliferation and tumor growth.

**Results:**

Using a GLI-responsive luciferase reporter assay, we show here that HH ligand could not mount a signaling response in the NSCLC cell line A549, even though Oxy186 and Oxy210 still inhibited non-canonical GLI activity and suppressed the proliferation of A549 cells. Further, we uncover an unexpected activity of these two oxysterols in inhibiting the WNT/β-catenin signaling at the level of LRP5/6 membrane receptors. We also show that in a subcutaneous xenograft tumor model generated from A549 cells, Oxy186, but not Oxy210, exhibits strong inhibition of tumor growth. Subsequent RNA-seq analysis of the xenograft tumor tissue reveal that the WNT/β-catenin pathway is the target of Oxy186 in vivo.

**Conclusion:**

The oxysterols Oxy186 and Oxy210 both possess inhibitory activity towards WNT/β-catenin signaling, and Oxy186 is also a potent inhibitor of NSCLC tumor growth.

**Supplementary Information:**

The online version contains supplementary material available at 10.1186/s13578-022-00857-9.

## Background

Non-small cell lung cancer (NSCLC) accounts for about 85% of all lung cancers and is the leading cause of cancer-related death worldwide [1]. Because most NSCLC patients are diagnosed at advanced stages and are usually not very responsive to conventional chemo- and radio-therapies, the treatment options for NSCLC are limited [2]. During the past decades, several targeted therapies have been developed for NSCLC, including those targeting mutations in EGFR, ALK, ROS1, BRAF and VEGF [3]; however, new treatments are still needed, especially for patients without such mutations. Developmental signaling pathways, such Hedgehog (HH) and WNT, play important roles in maintaining cancer stem cells. Their dysfunction often leads to cancer and underpins the reoccurring resistance to current therapies, causing cancer relapse [4, 5]. Targeting these developmental signaling pathways offers an alternative route toward developing novel treatments for advanced NSCLC.

HH signaling is initiated when the ligand-engaged receptor Patched1 (PTCH1) releases its repression on the transmembrane signal transducer Smoothened (SMO), thereby allowing SMO to traverse into the primary cilium and activate GLI transcription factors [6]. The WNT/β-catenin pathway is activated upon binding of WNT ligands (e.g. WNT1 or WNT3) to Frizzled (FZD) receptors and lipoprotein receptor–related proteins 5/6 (LRP5/6) co-receptors [7]. The LRP5/6 receptors then recruit Dishevelled (DVL) proteins to the plasma membrane, and in turn inactivate a destruction complex that contains the scaffold proteins AXIN and adenomatous polyposis coli (APC), as well as casein kinase 1α (CSNKA1) and glycogen synthase kinase 3β (GSK3B). This leads to the stabilization of the transcriptional co-activator β-catenin (CTNNB1), enabling it to enter the nucleus and form an active transcriptional complex with transcription factors of the lymphoid enhancer factor (LEF) and T-cell factor (TCF) families. Dysregulation of either the HH or WNT pathways frequently occurs in basal cell carcinoma, medulloblastoma, colorectal cancer and lung cancer [8, 9].

Oxysterols are derivatives of cholesterol that are formed naturally by oxidation, or can be synthesized in the laboratory using commercially available precursors (*eg.* pregnenolone) [10, 11]. These compounds are known to be either activators or inhibitors of the HH signaling pathway [12, 13]. Previously, we reported on two semisynthetic oxysterol derivatives, Oxy186 and Oxy210, that potently inhibit HH signaling downstream of SMO, possibly by inhibiting *GLI1* expression and/or GLI transcriptional activity in the HH pathway [11, 14]. In addition, Oxy210 showed propensity to inhibit TGF-β signaling by blocking the TGF-β type I receptor-mediated phosphorylation of downstream mediators SMAD2 and SMAD3 [11]. Both Oxy186 and Oxy210 are capable of inhibiting the proliferation of the NSCLC cell lines A549 and H2030 [11, 14], and we postulated that this activity was possibly due to suppression of HH/GLI signaling, which is known to play an important role in tumor growth associated with NSCLC [15]. In the present follow-up study, however, we found that A549 cells are not responsive to Sonic Hedgehog (SHH) ligand, suggesting that mechanisms other than canonical HH signaling might be at play. Here we show that Oxy186 and Oxy210 can also inhibit WNT/β-catenin signaling, most likely at the level of LRP5/6 receptors. In addition, Oxy186, but not Oxy210, also inhibits A549 xenograft tumor growth in vivo, and RNA-seq analysis of Oxy186-treated xenograft tumor samples confirmed the inhibition of WNT target gene expression, including *MYC* and *Cyclin D1* (*CCND1*). These results indicate that Oxy186 is a dual inhibitor of HH and WNT signaling, making it a potential candidate for developing new targeted therapies for NSCLC.

## Results

### A549 cells are not responsive to HH stimulation

**Fig. 1:**
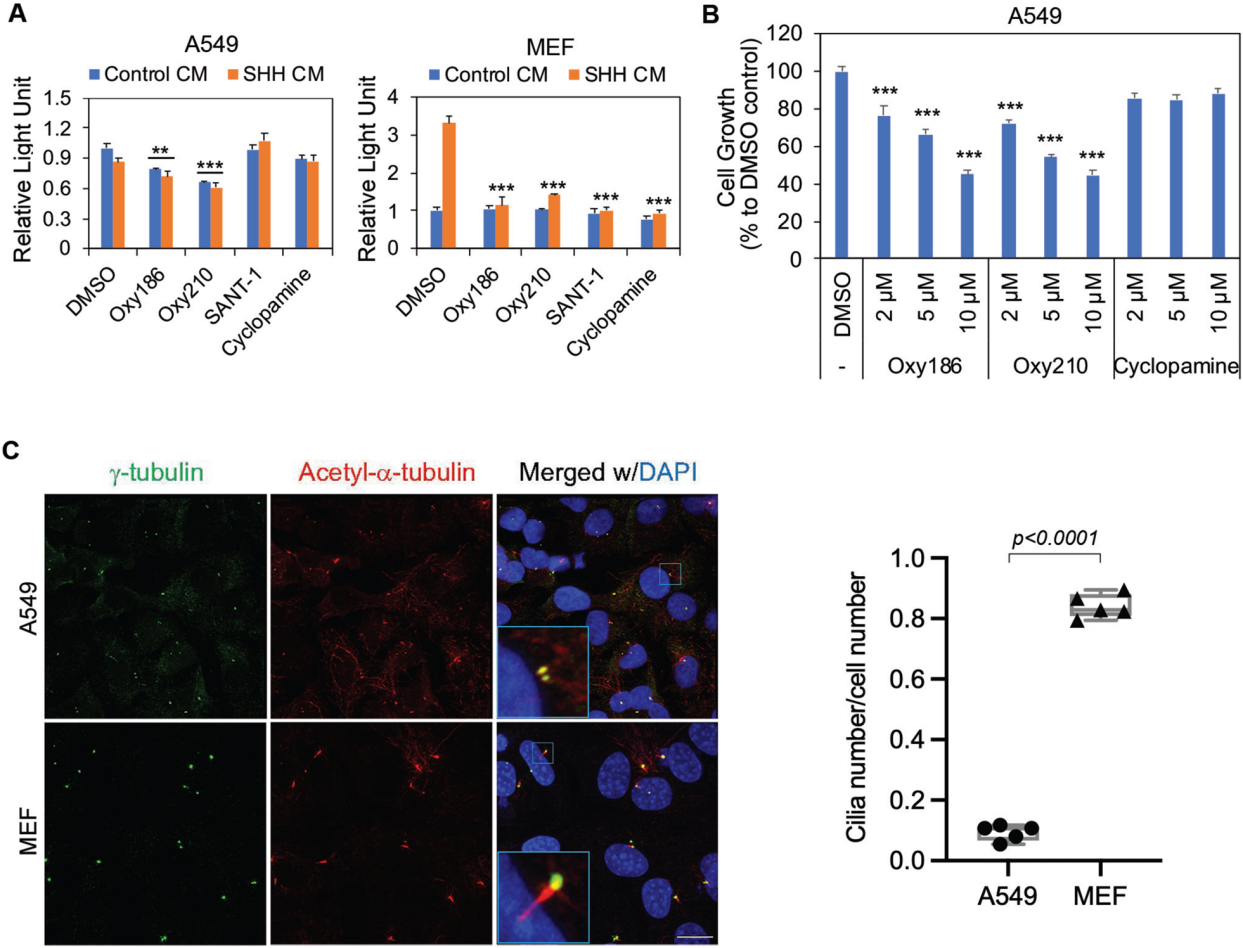
A549 cells are not responsive to SHH stimulation. **A**. Ligand-induced HH signaling takes place in MEFs, but not in A549 cells. A549 cells and MEFs transfected with 8xGliBS-Luc and pRK-TL were treated with DMSO, Oxy186, Oxy210 or the HH signaling inhibitors SANT-1 or cyclopamine, and stimulated with control CM or SHH CM. After 24 h, Firefly luciferase activity was measured and normalized to Renilla luciferase activity. Data from a representative experiment are reported as the mean of triplicate measurements ± SD (**p < 0.02, *** p < *0.001* vs. SHH CM-treated DMSO control). **B** Inhibition of A549 cell proliferation by Oxy186 and Oxy210, but not by HH/SMO inhibitor cyclopamine. A549 cell proliferation was measured after treating cells with the indicated compounds in DMEM containing 0.1% FBS for 2 days. Data from a representative experiment are reported as the mean of triplicate measurements ± SD (*** p < *0.001* vs. DMSO control). **C**. Primary cilia were only sparsely detected in A549 cells. Primary cilia were identified using immunofluorescence as acetylated-α-tubulin-positive axonemes proximal to γ-tubulin-positive basal bodies. Bar = 15 µm. The marked square areas are shown at higher magnification in the merged panels. Ratio of ciliated A549 cells or MEFs at 48 h after serum starvation are shown on the right. Data are reported as the mean from five different fields ± SD

We previously showed that Oxy186 and Oxy210 suppressed A549 cell proliferation, possibly by inhibiting GLI activity in the HH pathway [11, 14]. However, the transcriptional activity of a GLI-responsive luciferase reporter transiently introduced into A549 cells was not induced by treatment with conditioned media containing N-terminal SHH (SHH CM) (Fig. 1A, left panel). The same reporter can be activated in mouse embryonic fibroblasts (MEFs) by the same SHH CM (Fig. 1A, right panel), indicating that A549 cells are not responsive to HH stimulation. Consistent with the lack of HH signaling responsiveness, Oxy186, Oxy210 and two established HH/SMO inhibitors, SANT-1 and cyclopamine [16], did not inhibit HH signaling in A549 cells as they did in MEFs (Fig. 1A). Having found that A549 cells lack a HH signaling response, we repeated the previous cell proliferation assays [11, 14] and indeed found that the SMO inhibitor cyclopamine was unable to suppress A549 cell growth, but both Oxy186 and Oxy210 strongly suppressed cell growth (Fig. 1B). Since the presence of primary cilia is indispensable for HH signaling in mammalian cells [17], we examined the formation of primary cilia in over-confluent A549 cells and MEFs. Consistent with the lack of HH signaling responsiveness, primary cilia were only sparsely detected in A549 cells compared to MEFs (Fig. 1C).

### Oxy186 and Oxy210 suppress A549 cell proliferation through non-canonical GLI, and WNT/β-catenin pathways.

**Fig. 2:**
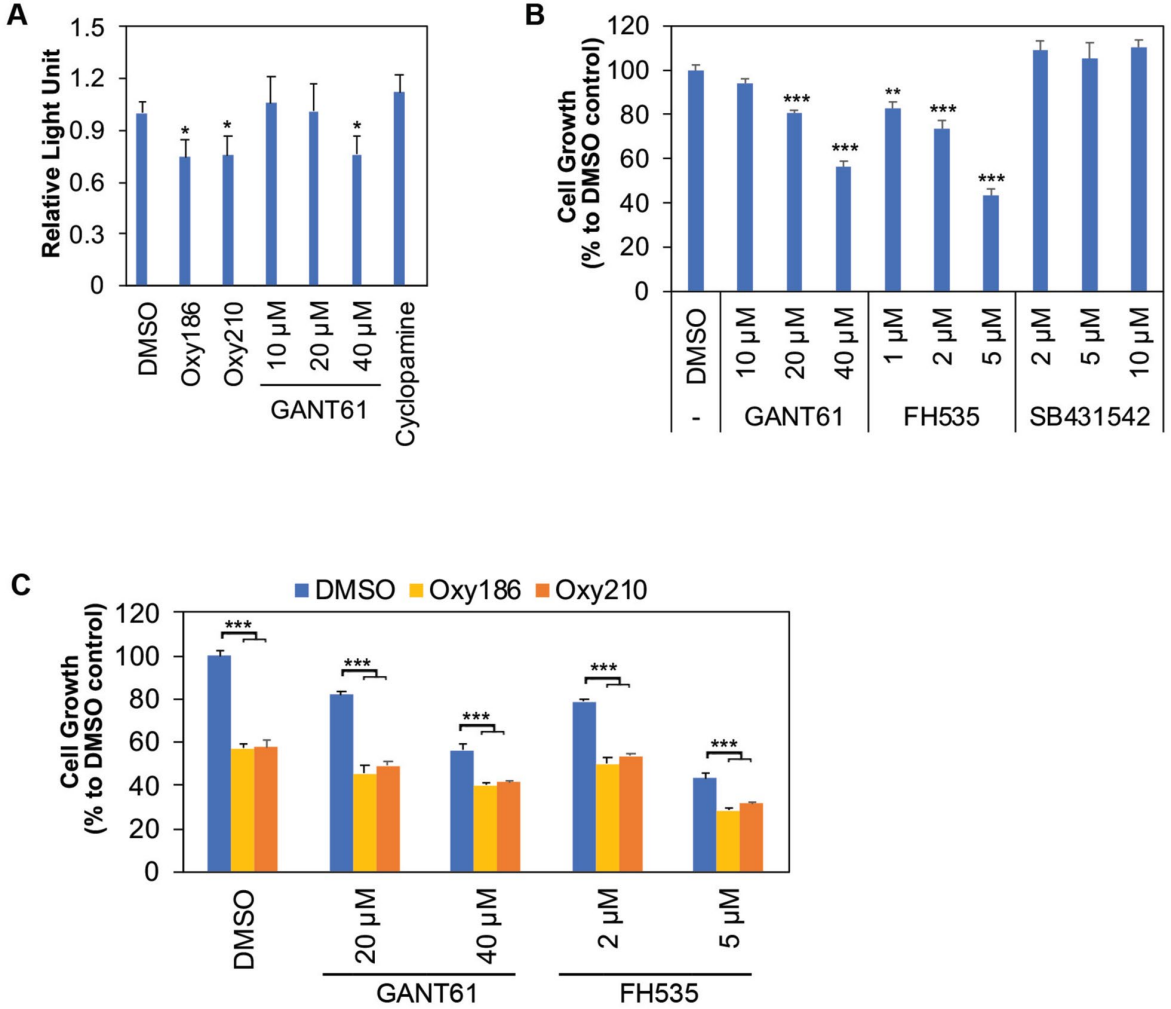
Oxy186 and Oxy210 inhibit A549 cell proliferation through non-canonical GLI and WNT/β-catenin pathways. **A**. Basal 8xGliBS-Luc reporter activity in A549 cells treated with Oxy186, Oxy210, the GLI inhibitor GANT61, or the SMO inhibitor cyclopamine. Data from a representative experiment are reported as the mean of triplicate measurements ± SD (*p < *0.05* vs. DMSO control). **B**. GLI inhibitor GANT61 and WNT pathway inhibitor FH535, but not TGF-β type I receptor inhibitor SB431542, inhibit A549 cell proliferation. Data from a representative experiment are reported as the mean of quadruplicate measurements ± SD (**p < 0.02; ***p < *0.001* vs. DMSO control). **C**. Oxy186 (10 µM) and Oxy210 (10 µM) further inhibited A549 cell growth in the presence of GLI inhibitor GANT61 or WNT pathway inhibitor FH535. Data from a representative experiment are reported as the mean of quadruplicate measurements ± SD (***p < *0.001)*

Besides the canonical, ligand-induced HH signaling, SMO-independent, non-canonical ways to activate GLI have been reported in many cancers [18]. A549 cells were previously reported to harbor high GLI activity despite their lack of responsiveness to SMO inhibitors [19]. Consistent with these findings, at the highest concentration tested (40 µM), the GLI inhibitor GANT61 [20] was able to suppress the basal activity of the GLI-luciferase reporter in A549 cells in the absence of HH ligand stimulation (Fig. 2A). Oxy186 and Oxy210 were also able to suppress the GLI-luciferase reporter to the same extent as GANT61 (Fig. 2A). Furthermore, GANT61 inhibited A549 cell growth in a dose-dependent manner (Fig. 2B), indicating that A549 cells are subject to growth inhibition of clamping down GLI activities, which is in line with our previous report [11, 14]. Unexpectedly, FH535, an inhibitor of WNT/β-catenin signaling [21], also strongly suppressed A549 cell growth (Fig. 2B), and both Oxy186 and Oxy210 could further inhibit A549 cell growth even in the presence of the GLI inhibitor GANT61 or the WNT/β-catenin inhibitor FH535 (Fig. 2C). These results suggest that these two oxysterols probably act on both the GLI and WNT/β-catenin pathways. Since Oxy210 was also previously reported as an inhibitor of TGF-β signaling [11], we tested the effect of SB431542, a well-known inhibitor of TGF-β type I receptors [22], on A549 cell proliferation. The results clearly showed that SB431542 does not suppress A549 cell growth (Fig. 2B), indicating that the mechanism whereby Oxy210 suppresses cell growth in A549 cells is not through inhibition of TGF-β signaling.

### Oxy186 and Oxy210 inhibit WNT/β-catenin signaling.

**Fig. 3:**
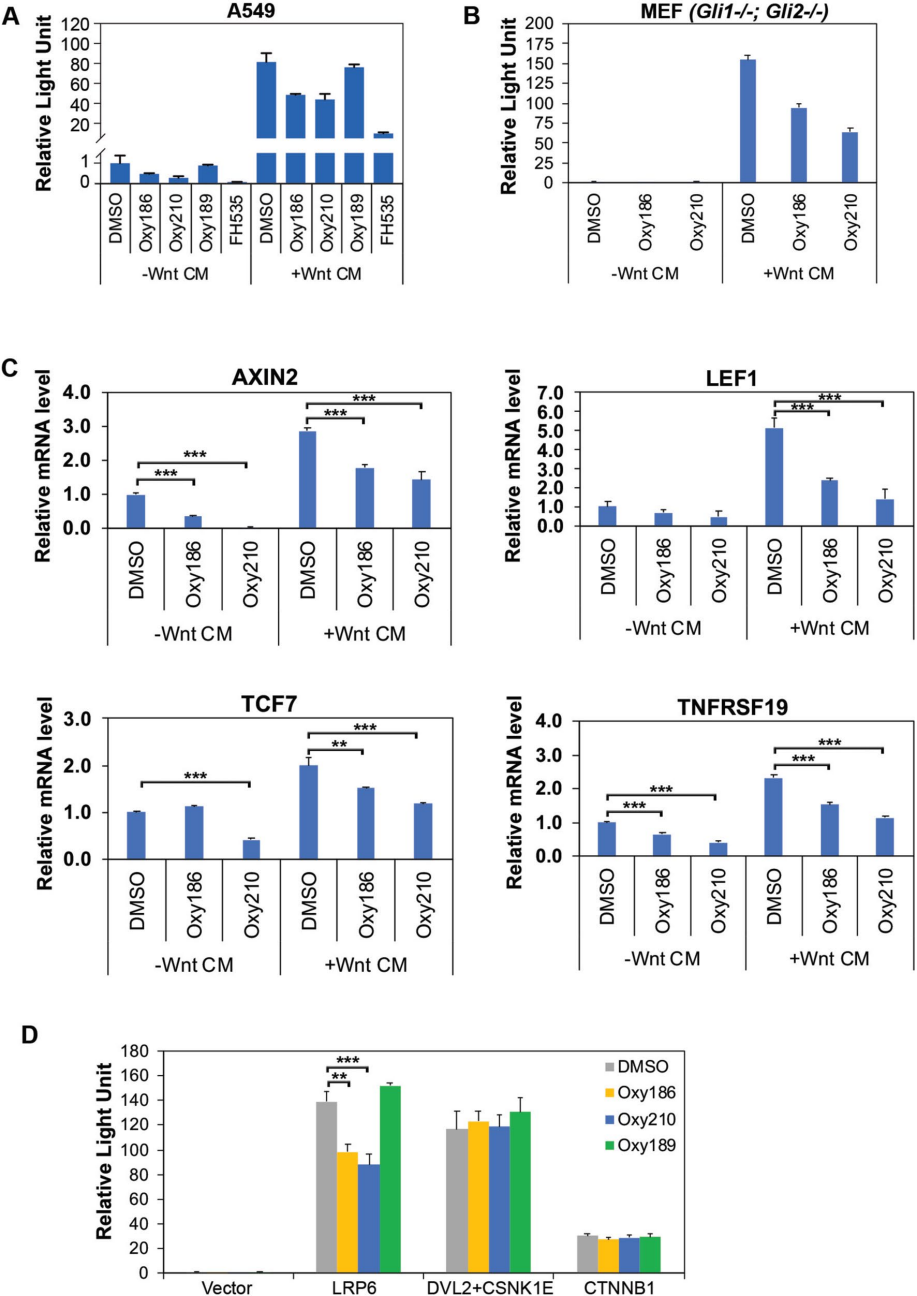
Oxy186 and Oxy210 inhibit WNT signaling in A549 cells. **A**. Oxy186 and Oxy210 inhibit WNT reporter (TOPflash) luciferase activity in A549 cells. A549 cells transfected with TOPflash and pRL-TK were treated with the indicated compounds in the absence or presence of WNT CM. After 24 h, Firefly luciferase activity was measured and normalized to Renilla luciferase activity. Oxy186 and Oxy210 inhibited WNT signaling, while the control Oxy189 did not. The WNT pathway inhibitor FH535 is shown as a positive control. Data from a representative experiment are reported as the mean of triplicate measurements ± SD. **B**. Oxy186 and Oxy210 inhibit WNT reporter luciferase activity independent of GLI activities. TopFlash luciferase assays were performed as above. Oxy186 and Oxy210 inhibit WNT CM-induced TOPflash luciferase activity in *Gli1*^*−/−*^;*Gli2*^*−/−*^ MEFs. **C**. Oxy186 and Oxy210 inhibit WNT pathway target gene expression. A549 cells were cultured in the absence or presence of WNT CM for 24 h, RNA was extracted and analyzed by RT-qPCR for the expression of the indicated genes and normalized to *RPS18* expression. Data reported as the mean (n = 5) ± SD (*** p < *0.001*; ** p < *0.02*). **D**. Oxy186 and Oxy210 inhibit TOPflash luciferase activity induced by expression of the WNT co-receptor LRP6, but not by expression of a combination of DVL2 and CSNK1E, or by CTNNB1. A549 cells were transfected with TOPflash and pRL-TK as well as the indicated plasmids, and treated with Oxy186, Oxy210 or control Oxy189 for 24 h before harvesting for luciferase assays. Data from a representative experiment are reported as the mean of triplicate measurements ± SD (***p < 0.001; **p < *0.02*)

To test if Oxy186 and Oxy210 possess WNT pathway inhibitory activities, we performed the WNT-dependent TOPflash reporter assay in A549 cells and found that WNT3A conditioned medium (WNT CM) induced a robust increase in luciferase activity, which was inhibited by FH535 as expected (Fig. 3A). Both Oxy186 and Oxy210 significantly inhibited the luciferase reporter activity, but Oxy189, another oxysterol derivative previously used as a negative control, did not (Fig. 3A), suggesting that the WNT inhibitory activity is specific to Oxy186 and Oxy210. Moreover, Oxy186 and Oxy210 were able to inhibit WNT3A-induced TOPflash activity even in a cell line in which both *Gli1* and *Gli*2 genes were knocked out (Fig. 3B), indicating that the inhibitory role of Oxy186 and Oxy210 on WNT signaling is independent of their ability to inhibit GLI signaling.

To corroborate results from the luciferase reporter assay, we examined the expression levels of several WNT target genes in A549 cells, namely *AXIN2*, *LEF1*, *TCF7* and *TNFRSF19*, by real-time quantitative PCR (RT-qPCR). As shown in Fig. 3C, WNT CM induced the expression of all four genes, and both Oxy186 and Oxy210 inhibited this effect.

During WNT/β-catenin signaling, binding of extracellular WNT ligands to the membrane-bound FZD and LRP5/6 receptors results in the signaling transduction through DVL [7], which in cooperation with casein kinase 1ε (CSNK1E) promotes the accumulation of CTNNB1 to elicit transcriptional responses [23, 24]. To determine at which step of this signal transduction cascade Oxy186 and Oxy210 exert their inhibition, we tested the ability of these two compounds to modulate the induction of TOPflash activity by overexpression of LRP6, a combination of DVL2 and CSNK1E, or CTNNB1 in A549 cells. In this setting, Oxy186 and Oxy210, but not the negative control oxysterol Oxy189, inhibited the TOPflash activity induced by LRP6; however, Oxy186 and Oxy210 were not able to inhibit that induced by DVL2 and CSNK1E or by CTNNB1 (Fig. 3D), suggesting that Oxy186 and Oxy210 likely work at the receptor level.

### Differential inhibition of xenograft tumor growth by Oxy186 and Oxy210

**Fig. 4:**
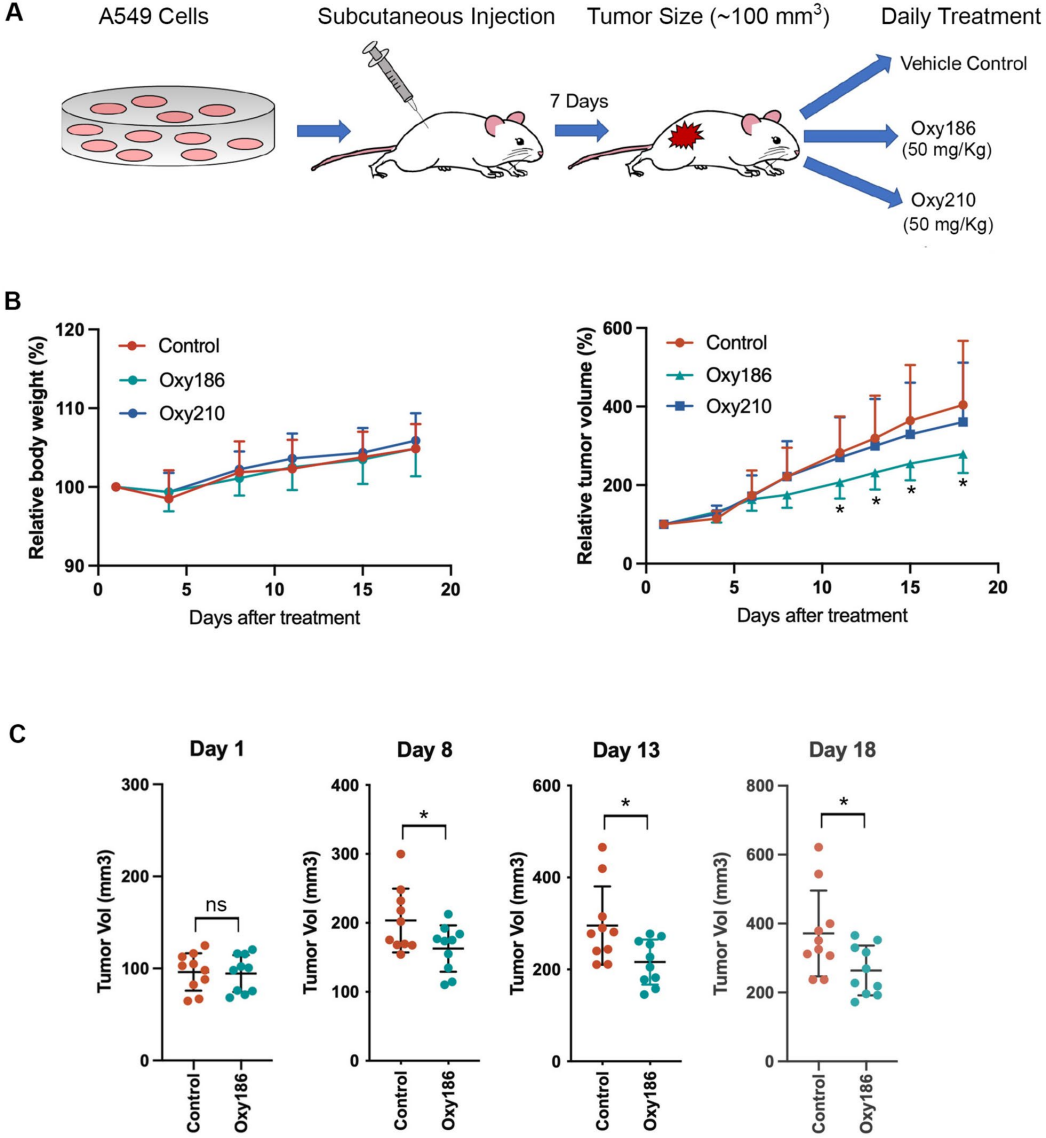
Inhibition of A549 cell-derived xenograft growth by Oxy186. **A**. Scheme of A549 cell-derived subcutaneous xenograft and subsequent treatment with Oxy186 or Oxy210. **B**. Relative animal body weight (body weight normalized to day 0 of oxysterol treatment) and relative tumor volume (tumor volume normalized to day 0 of oxysterol treatment). Data reported as the mean of 10 animals (n = 10) ± SD (*p < *0.05* vs. control). **C**. Absolute tumor volume of control and Oxy186-treated animals (n = 10) at day 1, day 8, day 13 and day 18 of treatment (*p < *0.05* vs. control)

To examine the efficacy of the oxysterols in inhibiting tumor growth in vivo, we tested Oxy186 and Oxy210 in a subcutaneous xenograft tumor model generated from A549 cells in nude mice. After the tumor size reached an average of ~ 100 mm^3^, the compounds were administered daily via oral gavage at a dose of 50 mg/kg (Fig. 4A). This treatment regimen was well tolerated, as mice in all 3 randomized experimental groups maintained similar body weight throughout the 18 days of treatments (Fig. 4B, left panel), during which we were able to complete data collection on all experimental mice. From the relative tumor volume data (Fig. 4B, right panel), we found that Oxy186 started to inhibit tumor growth at day 8, and significantly reduced the relative tumor volume from day 11 to day 18 of treatment, compared to that of the vehicle control. Surprisingly, Oxy210 did not show such inhibitory effect, and its effect on tumor growth was indistinguishable from that of the vehicle control (Fig. 4B, right panel). The absolute tumor volumes were also significantly reduced in the Oxy186 treatment group starting at day 8 (Fig. 4C).

### Basal cell carcinoma pathway is enriched in differentially expressed genes from Oxy186-treated xenografts

**Fig. 5:**
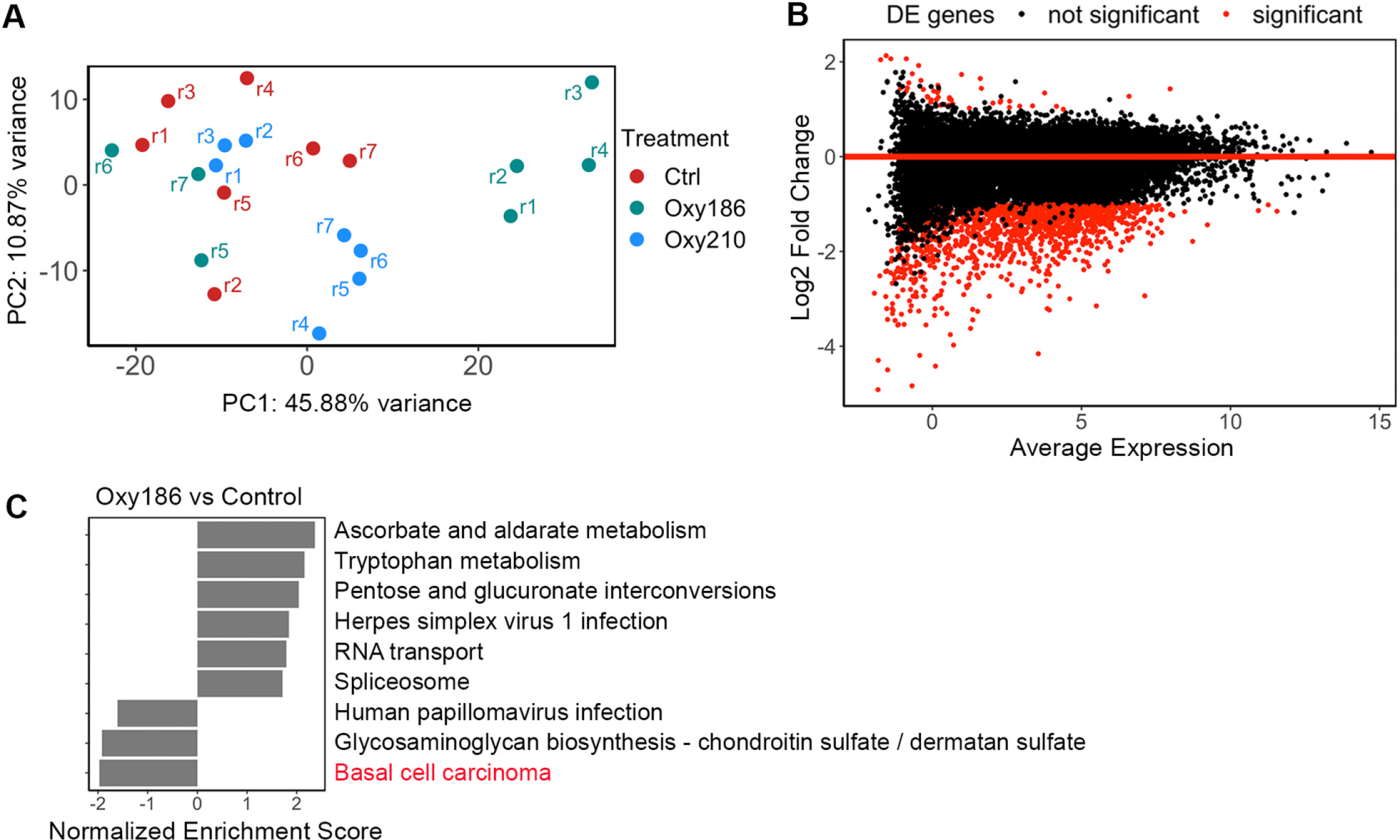
RNA-seq analysis of A549-derived xenografts. **A**. Principal component analysis plot of samples from RNA-seq analysis of control, Oxy186-treated, and Oxy210-treated xenografts. Bulk RNAs from seven samples (r1-r7) of each group were sequenced. Oxy186-treated sample r1-r4, but not r5-r7, showed a significant difference from the control samples. **B**. MA plot (log2 fold-change versus normalized expression) of differentially expressed (DE) genes in RNA-seq analysis of Oxy186-treated samples compared to control samples. Red dots: significantly DE genes (Fold > 2; *P*_*adj*_ ≤ 0.05). **C**. KEGG pathway enrichment analysis of affected genes comparing RNA-seq data from Oxy186-treated vs control xenografts. Cut-off: *P*_*adj*_ ≤ *0.05*

To determine which molecular pathways are targeted by Oxy186 to exert its tumor-suppressing activity, we extracted RNA from control, Oxy186-treated and Oxy210-treated xenografts and performed RNA-seq analyses. Consistent with the lack of inhibitory effect in tumor growth, Oxy210-treated samples did not separate from the control group on the principal component analysis plot (Fig. 5A). Out of the seven Oxy186-treated samples, four were clearly distinct from the rest of samples while three were mixed with the control group, indicating that these samples had different responses to the Oxy186 treatment (Fig. 5A). Using these four samples for further analysis, we found that more than 1700 genes were significantly downregulated while only 142 genes were significantly upregulated by Oxy186 treatment (Fig. 5B). From those differentially expressed genes, KEGG [25] pathway enrichment analysis identified 9 pathways that were significantly enriched (*P*_*adj*_ < 0.05), including the basal cell carcinoma pathway (Fig. 5C).

**Fig. 6:**
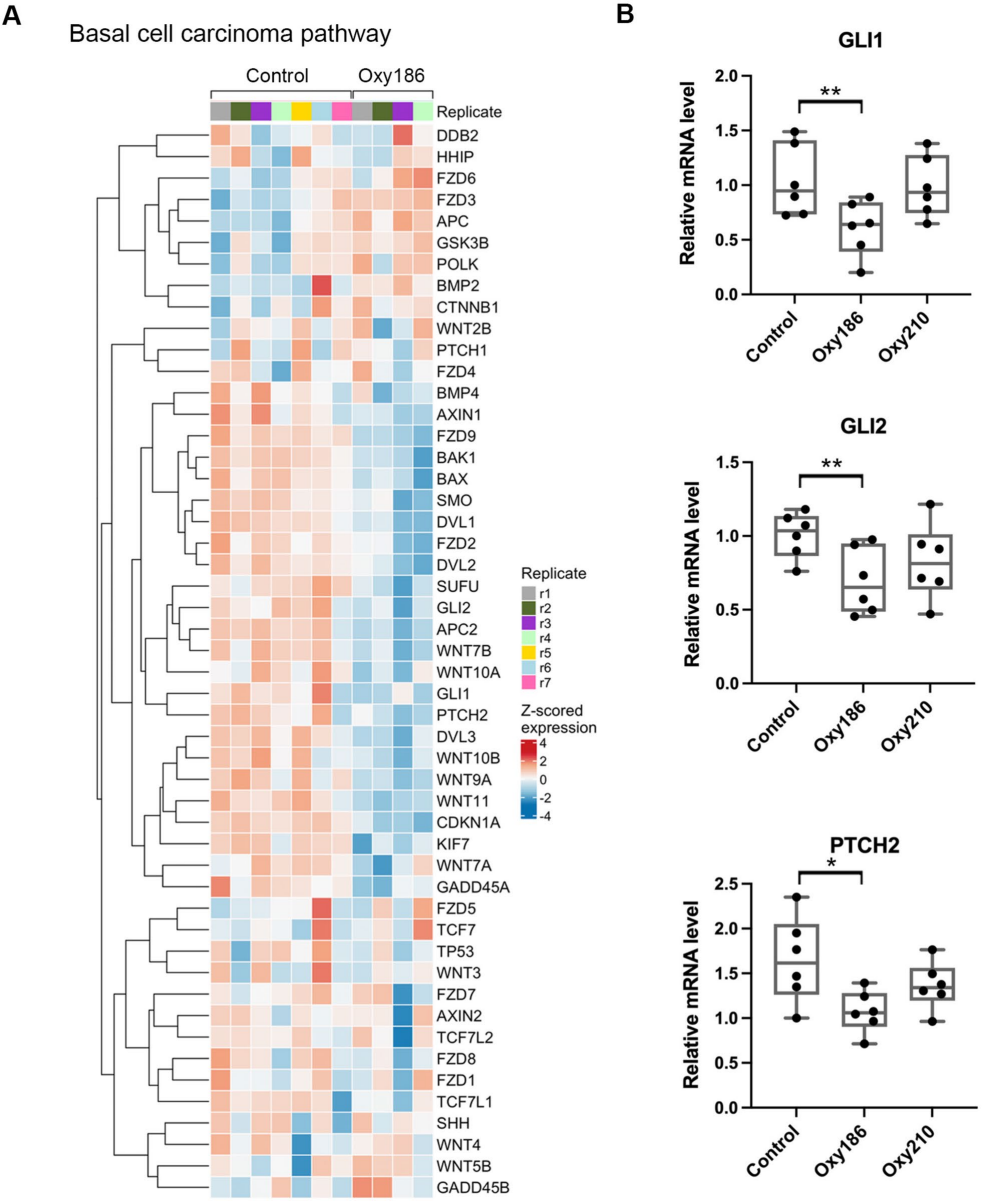
Oxy186 inhibits expression of many genes involved in the basal cell carcinoma pathway. **A**. Heat map of Basal Cell Carcinoma pathway. Gene expression in the heat map is standardized by row. **B**. RT-qPCR showed that Oxy186 significantly inhibited *GLI1*, *GLI2* and *PTCH2* expression in xenografts, whereas the effect of Oxy210 was not statistically significant. Data reported as the mean (n = 6) ± SD (* p < *0.05*; ** p < *0.02*)

Basal cell carcinoma is a common type of skin cancer derived from basal cells of the epidermis. Susceptible gene pathways known to affect basal cell carcinoma include the HH and WNT pathways [26] (Fig. 6A). Based on RNAseq data, we identified several genes in the HH pathway that were downregulated in Oxy186-treated samples compared to the control xenografts (Fig. 6A). We further confirmed downregulation of *GLI1*, *GLI2* and *PTCH2* in Oxy186-treated xenografts using RT-qPCR (Fig. 6B), but expression of some other HH target genes (e.g. *SHH*, *PTCH1*, *HHIP*) were not significantly affected by Oxy186 treatment (Fig. 6A, data not shown).

RT-qPCR analysis also showed a decreasing trend of *GLI2* and *PTCH2* expression in Oxy210-treated samples; however, no statistically significance could be assigned comparing to those of the control group (Fig. 6B). Because Oxy210 was found previously to inhibit TGF-β pathway [11], we measured mRNA levels of several known TGF-β target genes, namely *JUNB*, *ATF3* and *SMURF2*, using Oxy210-treated samples. Similar to the levels of *GLI2* and *PTCH2* expression in Oxy210-treated samples, these three genes were also downregulated in the Oxy210-treated xenografts, but the difference comparing to those of the control group did not reach statistical significance (Additional file 1: Fig. S1).

### RNA-seq analyses of xenograft tumors identifies the WNT pathway as a target of Oxy186

**Fig. 7:**
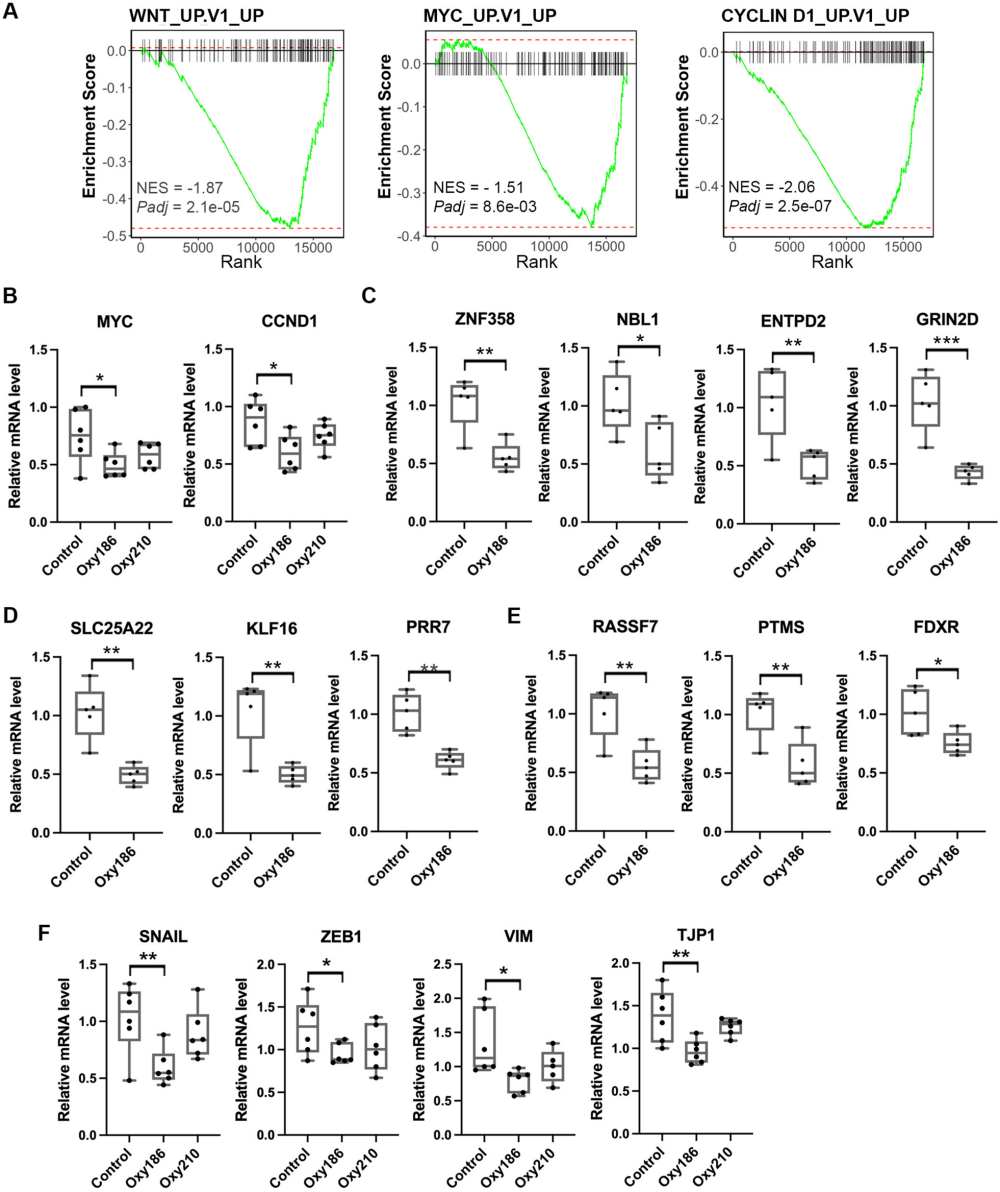
Oxy186 inhibits WNT pathway in xenografts. **A**. Gene Set Enrichment Analysis (GSEA) indicated that WNT upregulated genes, MYC upregulated genes and CCND1 upregulated genes were negatively enriched when comparing Oxy186-treated xenografts to controls. Graph shows the rank-ordered gene lists of the set. NES: normalized enrichment score. **B**. RT-qPCR confirmed Oxy186 inhibition of *MYC and CCND1 expression, but* Oxy210 treatment showed no statistical significance. Data reported as the mean (n = 6) ± SD (* p < *0.05*). **C**–**E**. RT-qPCR showed that Oxy186 treatment inhibited expression of WNT target genes **C**, MYC-induced genes **D**, and CCND1-induced genes **E**. Data reported as the mean (n = 5) ± SD (* p < *0.05*; ** p < 0.02; *** p < *0.001*). **F**. RT-qPCR showed that Oxy186 but not Oxy210 significantly inhibited expression of EMT-related genes in xenografts. Data reported as the mean (n = 6) ± SD (* p < *0.05*)

Using Gene Set Enrichment Analysis (GSEA) [27], we further identified 22 positively enriched and 49 negatively enriched gene sets in our dataset of Oxy186-treated xenografts compared to the oncogenic signature gene sets (C6) from the Molecular Signatures Database (MsigDB) [28]. Among the negatively enriched gene sets, several were related to the WNT pathway, including the WNT up-regulated gene set [29], the MYC up-regulated gene set [30], and the CCND1 up-regulated gene set [31] (Fig. 7A). *MYC* and *CCND1* are well known WNT-induced genes that promote cell proliferation and tumor growth [32]. We confirmed that expression of *MYC* and *CCND1* was indeed significantly downregulated in Oxy186-treated, and only slightly downregulated in Oxy210-treated xenografts (Fig. 7B). We then chose three or four genes from each gene set to validate their gene expression by RT-qPCR, and found that all of these genes were significantly downregulated in Oxy186-treated xenografts compared to their expression in control xenografts (Fig. 7C-E).

WNT/β-catenin signaling not only promote cell proliferation but also induce epithelial-mesenchymal transition (EMT) by inducing the expression of transcription factors, such as *SNAIL* and *ZEB1* [33, 34]. These two EMT-inducing transcription factors had been demonstrated to promote the invasive ability and bone metastasis of small cell lung cancer cells [35, 36]. We found that expression of these two genes as well as expression of *VIM* and *TJP1,* which encode mesenchymal marker Vimentin and ZO-1, respectively, were also significantly downregulated in Oxy186-treated Xenografts (Fig. 7F). While Oxy210 only had a slightly effect on these gene expression in xenografts (Fig. 7F). Taken together, these results indicate that Oxy186 targets the GLI and WNT pathways in xenografts.

## Discussion

The HH and WNT signaling pathways are known positive regulators of cell proliferation during stem cell self-renewal and oncogenesis. These two pathways are intrinsically linked and regulate each other’s transcriptional output in specific cellular contexts. A positive feedback loop between HH and WNT signaling takes place during epithelial transformation. On the one hand, GLI1 has been shown to stimulate WNT/β-catenin activity through SNAIL [37]. On the other hand, WNT signaling has been reported to induce *GLI1* expression, *GLI1* mRNA stabilization and GLI1 transcriptional activity in several cancer cell types [37–39]. Despite this reported cross-talk, we found that the inhibitory role of Oxy186 and Oxy210 on WNT signaling in A549 cells is independent of HH/GLI signaling activity, because both oxysterols can still inhibit WNT signaling in the presence of the GLI inhibitor GANT61, and even in a cell line in which both *Gli1* and *Gli2* were knocked out.

Although GLI1/2 are not activated in response to HH in A549 cells, both HH and WNT signaling could be activated in other NSCLC cell lines, or in other types of tumors (e.g., BCC, CRC and leukemia) [4]. In fact, it has been reported that the SMO inhibitor cyclopamine and the WNT inhibitor quercetin suppressed the growth of leukemia cells [40]. Simultaneous dysregulation of both the HH and WNT pathways in a tumor provides an advantage to neoplastic cells, which could influence relapse and drug responsiveness. The fact that Oxy186 inhibits both GLI and WNT signaling makes Oxy186 an attractive therapeutic candidate for targeting both pathways in multiple cancers.

Surprisingly, Oxy210 had no effect on A549 xenograft tumor growth despite that Oxy210 had lower IC50 concentration than Oxy186 in cell proliferation assays [11, 14]. In previous studies, we have shown that after oral delivery, both Oxy186 and Oxy210 have similar plasma exposure [11]. In addition, oral administration of Oxy210 was shown to have inhibitory disease modifying effects in a mouse model of NASH [41]. Therefore, the absorption of Oxy210 could not be the reason for its lack of activity in this study. However, since the levels of Oxy210 (or Oxy186) in the tumors were not measured, we cannot rule out that the absence of an Oxy210 effect on tumor growth was due to weak tumor penetration or reduced half-life in tumors. Secondly, the effect of Oxy210 in tumor growth could be cell context dependent. It will be interesting to determine the effects of these two oxysterols using different NSCLC lines, especially those with different p53 or RAS mutant status.

EMT is known to associate with lung cancer metastasis and resistance to chemotherapy and targeted drugs [42]. The fact that Oxy186 also inhibited expression of EMT-inducing transcription factor *SNAIL* and *ZEB1* as well as expression of mesenchymal marker *VIM* and *TJP1* suggests that Oxy186 could be also useful in treating the late stages of cancers, as those cancer cells are poised to undergo EMT, invasion and metastasis. It will be interesting for future studies to examine whether Oxy186 could inhibit tumor progression and metastasis in a preclinical metastasis model.

## Conclusion

In this report, we show that Oxy186 and Oxy210 can inhibit WNT/β-catenin signaling in addition to GLI signaling. We further demonstrate that Oxy186 inhibits A549-derived xenograft tumor growth and confirm the inhibitory effect of Oxy186 on WNT signaling in vivo. These results suggest an alternative strategy toward developing new treatments for advanced NSCLC.

## Materials and methods

### Cell culture, antibodies and reagents

The human lung cancer cell line A549 and mouse embryonic fibroblasts (MEFs) were cultured in Dulbecco's modified Eagle's medium (DMEM) supplemented with 10% fetal bovine serum (FBS). MEFs with *Gli1*^*−/−*^ and *Gli2*^*−/−*^ alleles were described [43]. Oxy186, Oxy189 and Oxy210 were provided by Max BioPharma. Monoclonal Anti-γ-Tubulin antibody, cyclopamine, SANT-1, GANT61, PEG400 and corn oil were purchased from Millipore Sigma (Burlington, MA). FH535 was obtained from Cayman Chemical (Ann Arbor, MI). SB431542 was obtained from Tocris Bioscience (Bristol, United Kingdom). Recombinant human TGF-β1 was obtained from PeproTech (East Windsor, NJ). Anti-acetylated α Tubulin Antibody (6-11B-1) Alexa Fluor^®^ 647 was purchased from Santa Cruz Biotechnology (Dallas, TX). Matrigel was obtained from BD biosciences (San Jose, CA). Dual-Luciferase^®^ Reporter Assay System was purchased from Promega (Madison, WI). N-SHH conditioned medium (SHH CM) and WNT3A conditioned medium (WNT CM) were prepared according to published protocols [44, 45].

### Luciferase assay

To measure HH or WNT signaling responses, A549 cells or MEFs were transfected using Lipofectamine 3000 (Thermo Fisher Scientific) with GLI response-element reporter (8xGliBS-Luc) plasmid or TCF reporter plasmid (TOPflash), respectively, and pTK-Renilla-Luciferase (pRL-TK) plasmid. 24 h following transfection, the cells were treated with SHH CM or WNT CM and the indicated compounds for another 24 h. Firefly and Renilla luciferase activities were measured using the Dual-Luciferase^®^ Reporter Assay System in a SpectraMax iD3 (Molecular Devices). The Firefly luciferase activities were normalized to the Renilla luciferase activities. Data are reported as the mean of triplicate measurements ± SD. Where indicated, A549 cells were also transfected with LRP6-pCS2 (a gift from Dr. Xi He, Addgene plasmid # 27,242), pCMV5-3xFLAG-DVL2 and pCS2-CSNK1E, or pEF-MYC-CTNNB1 (a gift from Dr. Henry Ho, University of California, Davis).

### Cell proliferation assay

A549 cells were plated on 96-well plates at a density of 2000 cells/well, and the cells were treated with the indicated compounds in DMEM/0.1% FBS for 48 h. Cell proliferation was measured using the Cell Counting Kit-8 (CCK8) (Dojindo Molecular Technologies) following the manufacturer’s protocol.

### Immunostaining for primary cilia

Overconfluent A549 cells or MEFs were serum-starved for 48 h before proceeding with immunostaining procedures. Briefly, the cells were washed with PBS and incubated with pre-treatment solution (0.5% Triton X-100, 0.4% paraformaldehyde in PBS) for 3 min. Then cells were fixed with 4% paraformaldehyde in PBS for 7 min, permeabilized with 0.5% Triton X-100 in PBS for 20 min and blocked with 5% Bovine Serum Albumin in PBST (0.05% Tween in PBS) for 30 min. After the blocking step, the cells were incubated with primary antibodies and secondary antibodies. γ-tubulin, acetylated α-tubulin and DAPI signal were captured with a Leica SP8 confocal microscope using Z-stack imaging. Primary cilia number/cell number was calculated from 5 images.

### RT-qPCR

Total RNA from A549 cells or from xenograft tissues was extracted with the RNeasy Plus Mini Kit (Qiagen) or the Direct-zol RNA Kit (Zymo research), respectively. Total RNA was reverse transcribed into cDNA with the High-Capacity cDNA Reverse Transcription Kit (Thermo Scientific). All PCR samples were prepared in triplicate wells in a 384-well plate and measured in a QuantStudio 5 Real-Time PCR System (Thermo Scientific). Primer pairs used were listed in Additional file 1: Table S1.

### Animal studies

The animal protocol was approved by the NCI at Frederick Animal Care and Use Committee. Resuspended A549-Luc2 cells (ATCC) were mixed with Matrigel at a 1:1 ratio and 100 μl suspension containing 1 × 10^7^ cells was injected subcutaneously in the hind flank of 6–8 weeks-old female athymic nu/nu mice (Charles River Laboratories). When the tumor size reached an average of 80–100 mm^3^, the mice were randomized into three groups with 10 mice in each group. Oxy186 or Oxy210, formulated in 3% DMSO + 7% Ethanol + 5% PEG400 + 85% corn oil, was administered to the mice once daily by oral gavage at a dose of 50 mg/kg. The control group was given the formulation solution. Tumor growth was measured three times weekly using a caliper, and absolute tumor volume was calculated by the formula (S x S x L)/2, where S and L were the short and long dimensions, respectively. Relative tumor volume was calculated by the formula 100× Tn/T0, where Tn = tumor volume of the tumor on day n, and T0 = tumor volume of the same tumor on day 0, when the treatment started. Mice were euthanized before the tumor size exceeded 2 cm in diameter.

### RNAseq analysis

Total RNA samples extracted from xenograft tumors with RNA integrity number (RIN) > 8.0 were sent for library preparation with the NEBNext UltraTM II RNA Librart Prep Kit and RNAseq was performed using an Illumina Novoseq 6000 instrument (Novogene). Raw reads were processed on the NIH Biowulf cluster. We removed adapter sequences, dropped leading/trailing low quality bases and short reads (< 25nt), and performed sliding window trimming with Trimmomatic version 0.39. A combined reference genome was created from Gencode GRCh38.p13 Release 37 and GRCm39 Release M26. Trimmed reads were aligned to the combined reference genome using STAR version 2.7.6a, and only read alignments mapped to the human reference genome were extracted for further processing. Gene expression was then quantified by summarizing the number of human reads uniquely mapped to each human gene using the featureCounts program from subread version 2.0.1. Downstream analysis was conducted in R version 4.0.3. We filtered out genes with low counts using the filterByExpr R function in the edgeR version 3.31.4 R package, and ﻿the trimmed mean of M-values (TMM) method was employed to normalize the raw counts using the calcNormFactors R function. The differential expression analysis was performed using the voom approach in the limma version 3.45.14 R package. Gene expression was measured using the log2 transformed counts per million (log2-cpm) values calculated from the cpm R function. The principal component analysis and heat map plots were generated based on the log2-cpm values. KEGG pathways [25] were retrieved using the KEGGREST version 1.29.0 R package, and the MSigDB C6 gene sets [28] were obtained from the msigdbr version 7.4.1 R package. The pre-ranked gene set enrichment analysis was conducted in the fgsea version 1.16.0 R package.

## Supplementary Information

**Additional file 1**: **Figure S1**. RT-qPCR showed that Oxy210 treatment inhibited expression of TGF-β target genes Data reported as the mean (n=6)±SD(p-value as indicated, not statitically significant. **Table S1**. Primers pairs for RT-qPCR

## Data Availability

The authors declare that all materials are available on request. The RNA-seq datasets used and/or analyzed during the current study have been deposited in the NCBI’s GEO with the accession number GSE199478.
