# Supplementary material for: Oxysterol derivatives Oxy186 and Oxy210 inhibit WNT signaling in non-small cell lung cancer
Source: Cell Biosci. 2022 Jul 30;12:119. doi: 10.1186/s13578-022-00857-9 (PMC9338492; doi:10.1186/s13578-022-00857-9)
Supplement: Supplementary file 1 — Additional file 1: Figure S1. RT-qPCR showed that Oxy210 treatment inhibited expression of TGF-β target genes Data reported as the mean (n=6)±SD(p-value as indicated, not statitically significant. Table S1. Primers pairs for RT-qPCR [file 13578_2022_857_MOESM1_ESM.pdf]

Supplemental Fig. S1

RT-qPCR showed that Oxy210 treatment slightly inhibited expression of TGF-ß target genes
Data reported as the mean (n = 6) ± SD (p value as indicated, not statitically significant).

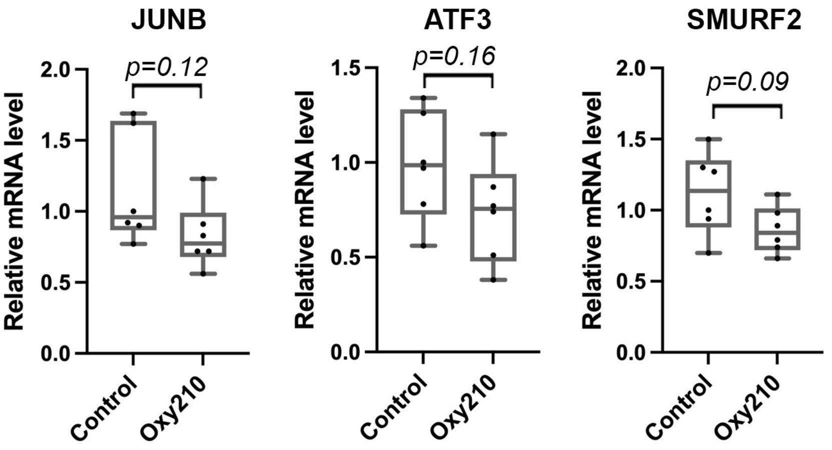

Supplemental Table S1: Primers pairs for RT-qPCR

	Forward	Reverse
h-JUNB	ACAAACTCCTGAAACCGAGCC	CGAGCCCTGACCAGAAAAGTA
h-ATF3	CGTGAGTCCTCGGTGCTC	GCCTGGGTGTTGAAGCAT
h-SMURF2	ACAGGAGGACAAGTTGTGGACT	GAGGCGGTTCTCCTTTCTTC
h-AXIN2	GTCCAGCAAAACTCTGAGGG	CTGGTGCAAAGACATAGCCA
h-LEF1	AGAACACCCCGATGACGGA	GGCATCATTATGTACCCGGAAT
h-TCF7	TTGATGCTAGGTTCTGGTGTACC	CCTTGGACTCTGCTTGTGTC
h-TNFRSF19	GACCTCAGCTCCACGAATATG	CACCCCACAACCAAGAGTCG
h-GLI1	GAAGTCTGAGCTGGACATGC	CCAACGGCAGTCAGTTTCAT
h-GLI2	CTCGCTAGTGGCCTACATCA	GTGTGTGTCCAAAGGCTGAC
h-PTCH2	CTCTGTAGGCATTGGCGTTG	AGTCAAAGTGGGAACCAGCA
h-Myc	GGCTCCTGGCAAAAGGTCA	CTGCGTAGTTGTGCTGATGT
h-CCND1	GCTGCGAAGTGGAAACCATC	CCTCCTTCTGCACACATTTGAA
h-ZNF358	ACGTGGACCCCAGCTATGAA	CGAGGTCGAAACTCGAAGACAT
h-NBL1	CATGTGGGAGATTGTGACGCT	CCTCGTGACTAGGCTCCTTG
h-ENTPD2	AGACAAGGAGAACGACACAGG	AGGCATCCAACAAGACTCTGG
h-GRIN2D	GAGGAAAGGCCGTTTGTCATC	TGTGGGTTCGGTTGAGCTG
h-SLC25A22	GCCAGCCAAGCTCATCAATG	GAGGCAGTCGGACATGCTC
h-KLF16	CAAGTCCTCGCACCTAAAGTC	AGCGGGCGAACTTCTTGTC
h-PRR7	ACCACCGTGTTACGAAGAGG	GGAAGGGCGTGACGATCTTG
h-RASSF7	TCTGTGGGGTCTCAGAGCAG	TGCGCCTCAGGACAAACTG
h-PTMS	ATGTCGGAGAAAAGCGTGGAG	CTGTCTTCTGCCGTTTGGGA
h-FDXR	CTGAGGCAGAGTCGAGTGAAG	CCCGAAGCTCCTTAATGGTGA
h-SNAIL	TCGGAAGCCTAACTACAGCGA	AGATGAGCATTGGCAGCGAG
h-ZEB1	GATGATGAATGCGAGTCAGATGC	ACAGCAGTGTCTTGTTGTTGT
h-VIM	AGTCCACTGAGTACCGGAGAC	CATTTCACGCATCTGGCGTTC
h-TJP1	CAACATACAGTGACGCTTCACA	CACTATTGACGTTTCCCCACTC
h-H2AFZ	GCAACTTGCTATTCGTGGAGATG	CAGGCATCCTTTAGACAGTCTTC
h-RPS18	GATATGCTCATGTGGTGTTG	AATCTTCTTCAGTCGCTCCA
